# Memory Disorders Related to Hippocampal Function: The Interest of 5-HT_4_Rs Targeting

**DOI:** 10.3390/ijms222112082

**Published:** 2021-11-08

**Authors:** Candice M. Roux, Marianne Leger, Thomas Freret

**Affiliations:** 1 CHU Caen, INSERM, UNICAEN, Normandie University, COMETE, CYCERON, 14000 Caen, France; candice.roux@unicaen.fr (C.M.R.); marianne.leger@unicaen.fr (M.L.); 2 PORSOLT, 53940 Le Genest Saint-Isle, France

**Keywords:** 5-HT_4_Rs, serotonin, hippocampus, memory disorders, therapeutic target, synaptic plasticity, cognition

## Abstract

The hippocampus has long been considered as a key structure for memory processes. Multilevel alterations of hippocampal function have been identified as a common denominator of memory impairments in a number of psychiatric and neurodegenerative diseases. For many years, the glutamatergic and cholinergic systems have been the main targets of therapeutic treatments against these symptoms. However, the high rate of drug development failures has left memory impairments on the sideline of current therapeutic strategies. This underscores the urgent need to focus on new therapeutic targets for memory disorders, such as type 4 serotonin receptors (5-HT_4_Rs). Ever since the discovery of their expression in the hippocampus, 5-HT_4_Rs have gained growing interest for potential use in the treatment of learning and memory impairments. To date, much of the researched information gathered by scientists from both animal models and humans converge on pro-mnesic and anti-amnesic properties of 5-HT_4_Rs activation, although the mechanisms at work require more work to be fully understood. This review addresses a fundamental, yet poorly understood set of evidence of the potential of 5-HT_4_Rs to re-establish or limit hippocampal alterations related to neurological diseases. Most importantly, the potential of 5-HT_4_Rs is translated by refining hypotheses regarding the benefits of their activation in memory disorders at the hippocampal level.

## 1. Introduction

Memory impairments are a core symptom of a number of neurodegenerative diseases, such as Alzheimer’s disease (AD) [1] and Parkinson’s disease (PD) [2], but are also common to several psychiatric pathologies such as major depressive disorder (MDD) [3] and schizophrenia (SCZ) [4]. Whether or not this is the core symptom of these pathologies, alterations of memory function always have a severely disabling effect on a patient’s everyday life. Indeed, memory function is a fundamental process which allows human beings to adapt from previous experiences and to progressively construct their unique identity [5].

Unfortunately, memory impairments remain therapeutically poorly apprehended. Over the past 30 years, only four drugs were approved to treat cognitive disorders. Initially developed in the context of AD—as the most prominent neurodegenerative disorder—the application domain of these drugs was thereafter extended to a larger number of pathologies. Among these drugs, three are acetylcholine esterase (Ach-E) inhibitors and the last is an N-methyl-D-aspartate receptor (NDMA-Rs) antagonist [6]. Regardless of their mechanism of action, they all show a limited efficacy and tolerance profile, leading to insufficient medical benefit. This contrasts with the large number of new therapeutic drug candidates tested in the field of preclinical studies, some demonstrating promising results. In 2008, over 172 drug development failures were registered in the field of AD [6]. Further, the only drug approved since 2003 was approved only very recently, with a use restricted to the United States [7].

Initially on the market for the treatment of gastrointestinal pathologies, type 4 serotonin receptors (5-HT_4_Rs) ligands progressively earned a place in the sun as a promising therapeutic target for memory disorders [8,9]. Two years after their discovery in 1988 [10], 5-HT_4_Rs started becoming the focus of intensive research for central nervous system (CNS) disorders, with over 100 patents of synthesized 5-HT_4_Rs ligands registered by 2014 [9]. Even today, the modulation of 5-HT_4_Rs remains a strategy of interest in the struggle against cognitive dysfunctions associated with psychiatric and/or neurological diseases [9].

This review will first discuss the current knowledge on memory function by focusing on the hippocampus and its alterations during physiological and pathological aging. Then, through a comprehensive discussion of the role of 5-HT_4_Rs in hippocampal memory processes, the relevance of its pharmacological modulation as a future therapeutic strategy in memory disorders will be argued in a broad extent.

## 2. Episodic Memory Function and the Hippocampal Formation

From the second half of the 20th century, case studies of patients with amnesia, as well as the development of a large number of animal models with memory disorders, enabled major breakthroughs in the understanding of the brain memory system—or how the brain stores different kinds of information. The idea of the existence of different forms of memory stems from this wealth of clinical work and fundamental studies. Often viewed as the most sophisticated, episodic memory is characterized by the capacity to re-experience a past personal event, situation or experience in the context in which it originally occurred [11]. A characteristic feature of episodic memory resides in the ability to bind together various interrelated stimuli and their spatial, temporal and conceptual relationships, to build up coherent memory representations [12]. Unfortunately, episodic memory shows the largest degree of decline in age-related cognitive impairments such as in AD [1] or even in several psychiatric contexts, such as MDD [3]. This review will mainly focus on episodic memory impairments and on the key related brain structure, namely the hippocampus.

### 2.1. The Hippocampal Formation

Lying deep in the medial temporal lobe (MTL), the hippocampus sits at the top of a hierarchy of cortical systems in which later stages integrate information from previous ones. This allows it to build complex representations and to influence earlier stages of operations through back projections—the proper definition of episodic memory [11]. Such consideration fuels the broad consensus that the hippocampus and surrounding MTL structures play a critical role in the encoding and subsequent retrieval of new long-term episodic memories.

A turning point in cognitive neurosciences came from patient case studies with hippocampal damage. One of the most famous examples comes from the post-surgery follow-up of patient H.M. (Henry Molaison) that enabled the role played by the hippocampus in episodic memory to be highlighted [13]. Following these clinical observations, several animal models with lesions of distinct brain structures, notably the hippocampus, were developed. First in rodents [14,15] and then in a non-human primate species [16], all models highlighted the hippocampus as having a core role in memory function. Since then, the sheer number of studies performed in experimental models of amnesia has demonstrated the role of the hippocampus in episodic memory [17,18,19] and demonstrated its anatomo-functional specialization. Thus, the ventral (or anterior) and the dorsal (or posterior) part of the hippocampus (in rodents and primate, respectively) differ markedly in their afferences/efferences and consequently in their dedicated role [20]. The ventral hippocampus (VH) has robust efferent connections to the rostral hypothalamus and amygdala and is mostly involved in the emotional components of memory processes [21]. Hence the ventral part of the hippocampus attracts much of the work on memory impairment related to psychiatric disorders, such as anxiety-induced depression and post-traumatic stress disorder. Conversely, the dorsal hippocampus (DH) is mainly involved in spatial memory processing [22], with outputs primarily projecting to the dorsal lateral septum and the mammillary body [20]. Further, the discovery of place cells (within the CA1) [23]—which activate specifically when a person is in a precise location (spatial information)—reinforced the theory of an anatomo-functional segregation within the hippocampus.

### 2.2. The Hippocampal Formation Circuitry

Composed of three cyto-architectonically distinct regions, i.e., the dentate gyrus (DG), the subiculum and the cornus ammonis (CA) with its three subfields (CA1, CA2 and CA3), the hippocampal formation forms a trisynaptic loop. The entorhinal cortex (EC) is the major source of both input and output of information within the hippocampus [24].

**Figure 1:**
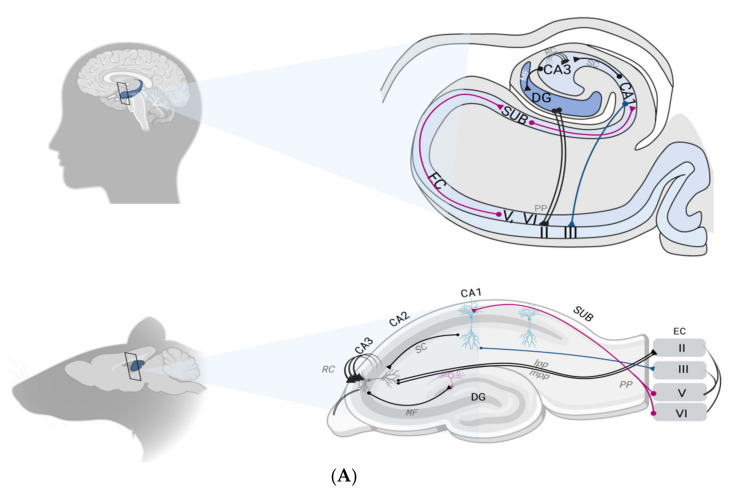

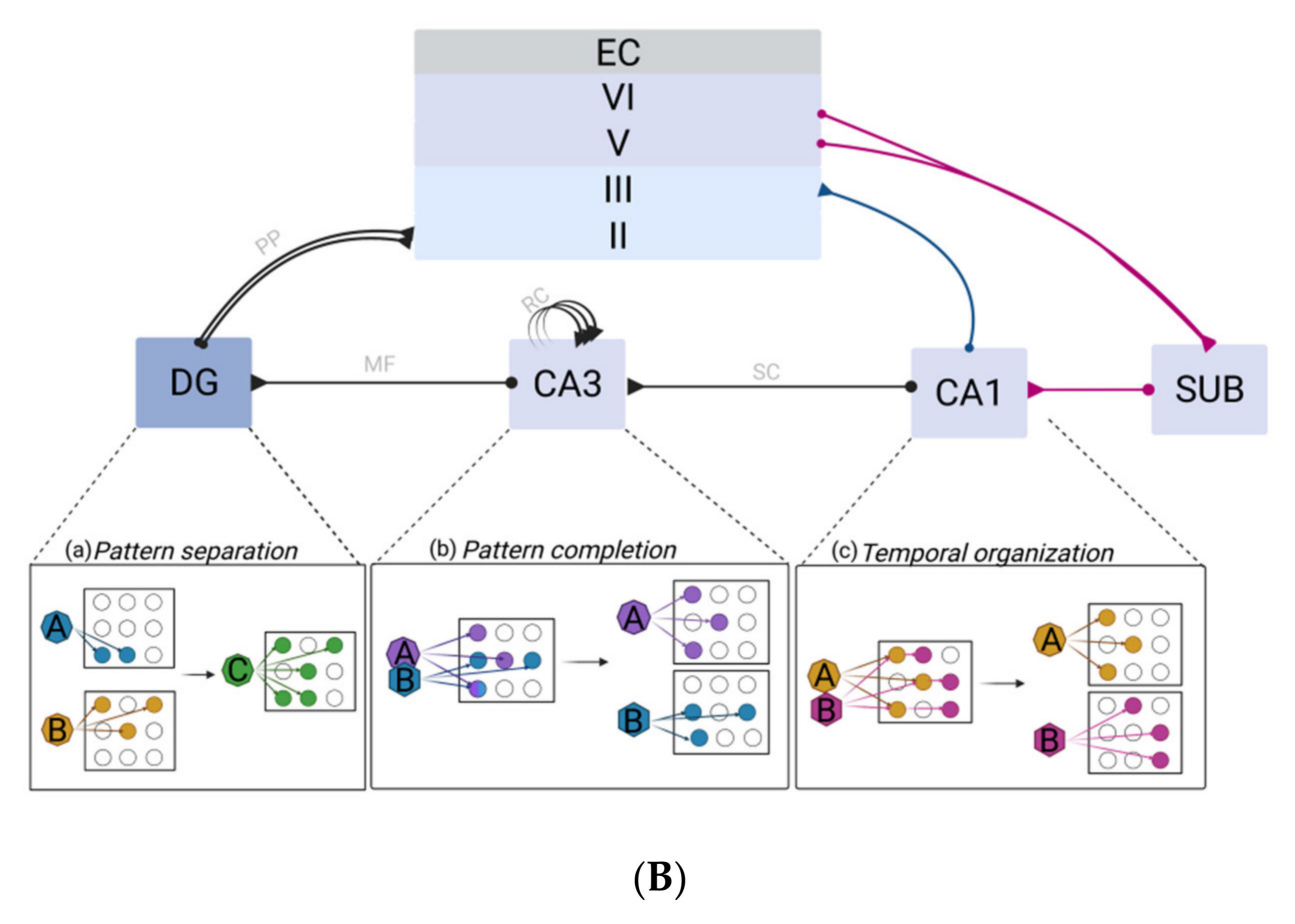
(**A**) Schematic representation of the location of hippocampal formation in both humans and rodents (left). Circuitry organization of the hippocampal formation in both species is depicted (right). Main inputs to the hippocampus are provided by superficial layers of the EC. Inputs converge to the CA1 through both the tri-synaptic pathway (DG, CA3 and CA1) and monosynaptic pathway, directly to the CA1 through the layer II of the EC. Recurrent collaterals (RC) of the CA3 contact other CA3 neurons and form the auto-associative network. The CA1 connection with the subiculum provides the main hippocampal outflow back to the deep layers of the EC (adapted from Small et al. 2011). (**B**). Representation of the functional specialization of each hippocampal subfield. The DG–CA3 axis is assigned to pattern separation (a), a function allowing it to disambiguate sensory inputs from similar experiences. Two similar inputs (A) and (B) are thus represented as two non-overlapping inputs. The pattern-separated signals from the DG are then projected onto the CA3 via the mossy fibers (MF) pathway. The CA3 is specialized in pattern completion (b), a process by which a partial or degraded subset (A) and (B) of the initial input can re-activate the retrieval of the whole context through a generalization process (C). The CA1 performs temporal organization of sequentially activated place cells (c). During spatial navigation, temporally close events (A→B) activate place cells in sequences that are then played out separately on a compressed time scale as a specific theta sequence (A/B). Abbreviations: CA1, CA3: cornus ammonis 1,3; DG: dentate gyrus; EC: entorhinal cortex; lpp, lateral perforant path; MF: mossy fibers; mpp, medial perforant path; PP, perforant path; SC, schaffer collateral pathway; RC: recurrent collaterals; SUB: subiculum.

Before being projected into the hippocampal formation through the EC, information may arise either from the parahippocampal gyrus or the perirhinal cortex, respectively encoding spatial and object representations. Mostly concentrated within the superficial layers (II-III) of the EC, this flow of information can reach the pyramidal neurons of the CA1 area by two distinct pathways. Indeed, the apical shafts of the CA1 area can be reached either directly (1/6 synapses) thus constituting the perforant path (PP), or using a tri-synaptic pathway, i.e., first passing by the DG, then the CA3 (through mossy fiber projections, MF), to finally reach CA1 through the Schaffer collateral pathway (SC). Finally, CA1 pyramidal neurons send their axons to the subiculum which flows information out to the EC, within its deep layers (V-VI) [24] (Figure 1A).

#### 2.2.1. The Dentate Gyrus–CA3 Pathway: Pattern Separation and Completion

The DG is renowned for its crucial role in the non-overlapping encoding of episodes presenting a high degree of similarity to limit interferences through a process known as pattern separation (PS) [24]. Compared with other hippocampal subfields, DG is more prone to representing highly similar scenes in a distinct fashion (Figure 1B). Therefore, damage to the DG leads to PS capacity impairment, both in humans and in animal models [25,26]. Hence, the DG acts as a competitive learning network precluding redundancy, where only the most relevant input patterns are selected among the continuous flow of information from diverse origins arising from the EC. Sparse recoding of EC inputs is achieved by keeping a low proportion (1 to 2%) of active DG excitatory granule cells (GC). This first selection stage is enabled by three main characteristics of the DG: the DG exhibits the highest densities of GABAergic interneurons compared with other subfields, thus providing strong inhibition to GC; GC have a low firing rate; and the GC receive outnumbered projections from the EC (from ~110,000 EC fibers, GC receive ~1.2 million inputs in each rat hippocampal hemisphere) [24].

The pattern-separated signals from DG are then projected onto the CA3 via the MF pathway, which constitutes the second selection stage. Indeed, MF synapses exhibit sparse but powerful connection to the CA3. Each CA3 cell receives ~50 MF inputs [24]. Such a method of projection favors a randomizing effect, since a set of neurons will be active for a unique event, leading thus to very different representations in the CA3 even for two highly similar events. This diluted connectivity substantially contributes to the final orthogonalization of the information which is essential to PS [24].

PS is fundamental, and any impairment of this capacity ultimately hampers the holistic retrieval of multidimensional episodes: the pattern completion. The aim of pattern completion is to enable the recall of a whole memory from partial cues (Figure 1B). This function mainly relies on the hippocampal CA3 network [27]. In fact, the unique presence of recurrent axon collaterals (EC) in CA3 neurons accounts for the highest number of synapses observed in CA3 pyramidal cells’ dendrites. The CA3 area is viewed as a single operating network (auto-associator), which allows arbitrary associations between inputs [24,27]. Subsequently, an event is represented by a set of concurrent neuronal firing, by which each feature can be re-activated by RC during recall [24,28].

Another particularity conferred by the RC to the CA3 region is its ability to act as an auto-attractor [24,27]. An auto-attractor maintains the steady firing of a set of neurons, which were first selectively activated during a specific task, particularly a spatial task. This function is fundamental since the probability of overlapping two events in a same space location is high. Further, the high plasticity of the CA3 area within a very short timing window is likely to allow rapid, “on the fly” encoding of information, thus facilitating associations between any spatial location [24,28].

#### 2.2.2. Multifaceted Roles of the CA1

Considered as the primary output of the hippocampus, the CA1 area performs accurate representation of a whole context, resulting from integrative computation between its two inputs [29]. This is supported by the generally assumed fact that direct input from EC is not sufficient to trigger an action potential in the CA1 by itself, but requires concurrent CA3 input. In line with a set of evidence regarding selective damage to the CA1, the discovery of sequence cells broadens the spectrum of functions that are assigned to this region with a role in the temporal aspects of memory [30] (Figure 1B). Accordingly, the double set of CA1 afferent would then allow CA1 cells to compare incoming information—corresponding to the currently occurring event—from the lateral EC, with information arising from the CA3 which represents familiar information [24,29]. In addition, back projections from the CA1 to the neocortex support the role of the CA1 in the memory consolidation process. Indeed, plasticity of CA3–CA1 synapses allows the whole episode encoded in CA3 to be represented in CA1 in longer term types of memory, than CA3. The CA1 can ensure an efficient recall by acting as a recorder of the recall activity of CA3 from a partial cue [29].

### 2.3. Synaptic Plasticity as a Correlate of Hippocampal Memory

The unraveling of mechanisms by which the hippocampus encodes and stores information has a long history. Efforts made over the past decades of research on this topic have progressively lead to the now widely accepted synaptic plasticity and memory hypothesis: “*activity-dependent synaptic plasticity is induced at appropriate synapses during memory formation, and is both necessary and sufficient for the encoding and trace storage of the type of memory mediated by the brain area in which that plasticity is observed”* [31]. Indeed, a defining characteristic of the hippocampus is this incredible ability to undergo activity-dependent functional and morphological remodeling via plasticity mechanisms. Over a century ago, Ramón y Cajal raised the idea that the dynamics of neural circuits (i.e., the changes in the efficacy of synapse transmission) would serve memory function. He was the first to propose the cellular theory of memory storage as an anatomical change in synaptic functional connections. This foreshadowed the Hebbian theory “*cells that fire together wire together”* that led to the assumption that associative memories are formed by synaptic plasticity, driven by temporal contiguity of pre- and post-synaptic activity [32]. This appealing cellular basis for learning and memory was further supported by the discovery of long lasting potentiation of synaptic strength, now known as long term potentiation (LTP). The characteristics of LTP (cooperativity, associativity, input specificity, as well as its durability) serve as non-trivial explanations for the great capacity, rapid acquisition and stability of memory [33].

Bliss and Lomo were the first to demonstrate the existence of LTP in the hippocampus following brief trains of high-frequency stimulation (HFS-100 Hz) [34]. Following this pioneering work, thousands of papers have been published on ex vivo hippocampal LTP, using different sets of stimulating protocols [35], such as theta-burst stimulation (TBS-5 Hz). As a matter of fact, the potentiation effects have a deep relationship with rhythmic bursts of activity that mimic naturally occurring brain oscillations [36]. Respectively described as gamma -γ- (30–100 Hz) rhythms for HFS and theta -θ- (4–12 Hz) rhythms for TBS, these oscillatory frequencies are observed during spatial and contextual learning [23,37,38]. Importantly, phase-amplitude coupling between theta and gamma oscillations has been reported across species, including mice, rats, and humans. Additionally, this phase-amplitude coupling is known to play a critical role in hippocampus-dependent memory processes [39].

Further, performance in hippocampal-dependent memory tasks has been associated with changes in LTP [40,41]. Inhibitors of hippocampal LTP were found to block both learning and retention when assessed in spatial memory tasks [40,41]. Additionally, several biochemical changes that occur after induction of LTP also arise during memory acquisition [42]. Since then, LTP has become a prototypical experimental model for the assessment of basic mechanisms involved in learning and episodic-like memory formation [35].

The induction of hippocampal LTP—in almost all of its subfields—is dependent on NMDA-Rs (with the exception of the MF-CA3 which can also display a form of LTP independent of NMDA-Rs) [42]. Therefore, the critical event leading to induction of LTP is the influx of calcium ions into the postsynaptic spine upon NMDA-Rs activation. Subsequent to calcium entry is the increase in calmodulin kinase II (CaMKII) activity that contributes to enhanced AMPA conductance and new addressing to the membrane. In addition, two other major pathways that involve different protein kinases, cyclic adenosine-monophosphate (cAMP)-dependent protein kinase (PKA) and extracellular regulated kinase (ERK), have also been identified as triggered by NMDA-Rs activation [42]. Downstream extracellular signals, including brain-derived neurotrophic factor (BDNF) have further been proposed to support the long lasting changes in synaptic function [42,43].

Nevertheless, hippocampal synaptic plasticity does not resume to LTP. Depotentiation (DP)—the reversal of LTP—and long term depression (LTD), which denotes the weakening of synapses, were also described in the hippocampus [43]. Both are necessary to specific forms of memory—also termed flexibility—that requires extinction of the obsolete memory traces, such as in the novelty recognition task [44]. These two synaptic plasticity processes are induced by low-frequency stimulation (LFS-1 Hz), which ranges around the hippocampal delta frequency band (0.5-4 Hz). Otherwise, both synaptic plasticity processes seem to rely upon similar mechanisms to LTP at a molecular level [45].

### 2.4. Neurotransmission Systems in the Hippocampus

Cellular events supporting learning and memory are the result of complex interactions between various neurotransmission systems. Most knowledge regarding these processes stems from the observation of the dysfunction of these systems in pathological conditions or from experiences of pharmacological manipulation [42,46]. The neurotransmitters and neuromodulators systems involved in hippocampal memory function are incontestably numerous. Therefore, this discussion is limited to those having a key pivotal role and/or having demonstrated a strong relationship with hippocampal serotonergic function [47], and more specifically with 5-HT_4_Rs, which are the core of this review.

#### 2.4.1. The Glutamatergic System

The excitatory amino acid glutamate is the most abundant amino acid transmitter in CNS and is largely involved in learning and memory. The hippocampus is comprised of 90% of glutamatergic cells and hence is enriched in glutamate receptors, mainly AMPA receptors (AMPARs) (α-amino-3-hydroxy-5-methyl-4-isoxazolepropionic acid receptor) and NMDA receptors (NMDARs) [48]. The major role played by these two ionotropic receptors, as well as by metabotropic receptors (mGluRs) [42], in synaptic plasticity (LTP process) and thus in memory formation, is widely accepted.

In rodents, pharmacological disruption of glutamatergic-mediated neurotransmission is accompanied by memory deficits in hippocampal dependent tasks (such as Morris water maze (MWM), passive avoidance, and radial maze tasks). In contrast, activation of glutamatergic transmission was related to improved memory performance [46]. Furthermore, the hippocampal atrophy in cognitively impaired patients, as well as the observed compensatory NMDARs’ over-activation (leading to excitotoxicity) also contributed to ascribing the glutamatergic system at the core of cognitive processes. Based on these observations, NMDARs antagonist-based therapies were proposed as an interesting strategy in AD (Memantine, MEM) [49] and MDD (Ketamine) [50]. However, despite its promising beneficial effects in preclinical studies, MEM showed poor clinical efficacy [49]. Of most interest, 5-HT_4_Rs have been shown to be expressed on glutamatergic neurons and consequently could be a target to modulate the glutamatergic system [51]. Hence, this constitutes an interesting avenue of research for the treatment of memory disorders.

#### 2.4.2. The GABAergic System

Memory function homeostasis relies on an intricate balance between excitatory and inhibitory transmission. γ-Aminobutyric acid (GABA) represents the major inhibitory neurotransmitter of the CNS. Although hippocampal GABAergic interneurons account for only 10 to 15% of the total neuron population, their large anatomical and functional diversity across all subfields of the hippocampus allows a powerful regulation of cellular and network activity [52]. Indeed, hippocampal GABAergic inputs arise from the medial septum (MS) and specifically innervate hippocampal GABAergic interneurons. Back projections to the MS form a reciprocal loop, which is considered to play a critical role in the generation of hippocampal rhythmic activity. Therefore, GABAergic interneurons strictly regulate both spatial and temporal extents of hippocampal activity, under a synchronized activity at theta frequency of neuronal populations. Moreover, both in vivo and in vitro studies have also underlined a role of GABAergic interneurons in driving gamma oscillations [52]. Finally, the bursts of population of pyramidal cells that occur during slow-wave sleep in sharp-wave ripples appeared related to an increase in DG interneurons firing [52].

**Figure 2:**
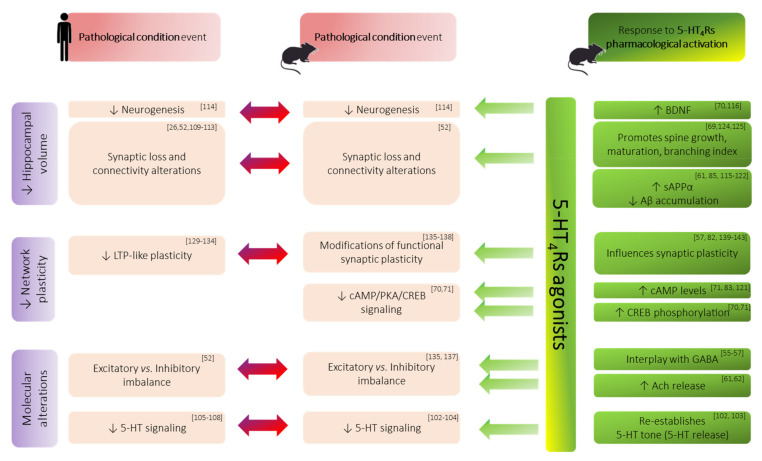
Summary of major hippocampal alterations (purple boxes) associated with memory impairments in both human and animal models of amnesic condition (red boxes). The beneficial effects of 5-HT_4_Rs pharmacological activation are represented at each level of alteration (green boxes). ↑ denotes an increase; ↓ denotes a decrease. Abbreviations: Aβ: beta-amyloid peptide; Ach: acetylcholine; BDNF: brain derived neurotrophic factor; cAMP: cyclic adenosine monophosphate; CREB: cAMP response element-binding protein; GABA: gamma-aminobutyric acid; LTP: long-term potentiation; PKA: protein kinase A; sAPPα: soluble alpha-amyloid precursor protein; 5-HT: serotonin; 5-HTR: serotonin receptor.

Whether memory improvements are supported by blockade or activation of GABA-receptors (GABARs) is often controversial, GABAergic neurotransmission clearly appears to be involved in memory function. The main source of these discrepancies may be due to the type of GABARs which are targeted. GABA_A_Rs are ionotropic receptors, permeable to chloride and mediate fast tonic inhibition on post-synaptic sites. Their activation is associated with altered memory performance [53]. Conversely, metabotropic GABA_B_Rs that are preferentially located on post-synaptic terminals, mediate slow phasic inhibition [52]. Their blockade may have beneficial effects on memory [53] and can modulate LTP [54]. Interestingly, application of the 5-HT_4_Rs agonist BIMU-8 was found to stimulate GABA release in guinea pig hippocampal slices [55,56] (Table 1, Figure 2). Additionally, through different conditioning protocols of LTP induction, the authors of this current study recently demonstrated an interplay between 5-HT_4_Rs activation and GABAergic neurotransmission within the hippocampal CA1 area [57]. This reinforces the interest of 5-HT_4_Rs as a modulatory target to treat memory disorders.

#### 2.4.3. The Cholinergic System

The hippocampus receives regulatory cholinergic inputs from the septal nuclei via the pre-commissural branch of the fornix. Cholinergic inputs are known to play an important role in hippocampal-dependent memory, either through nicotinic (ionotropic, mainly α7 sub-type) or metabotropic M1-M5 receptors [58].

Numerous humans and animal studies have linked acetylcholine (Ach) neurotransmission to learning and memory. Indeed, the increased release of hippocampal Ach during a memory task (notably spatial) was demonstrated to be positively correlated to improvements of learning performance [59]. Additionally, the administration of muscarinic receptor antagonists (atropine, scopolamine) induced cognitive impairments. Scopolamine is even considered as a gold standard in preclinical research to identify potential anti-amnesic properties of drug candidates. Therefore, high expectations have been placed on this neurotransmission system in the search for new drugs to treat memory disorders. Hence, three of the four drugs in the market to date (galantamine, rivastigmine, donepezil) aim to increase Ach levels by inhibiting the enzyme responsible for its degradation (AchE) [6].

Of most interest, Ach inputs mostly contribute to pacing intra-hippocampal theta rhythm [58]. This activity rhythm is critical to memory since it favors cellular excitability through the suppression of various potassium currents. Additionally, the cholinergic system is particularly influential in its interaction with the neuro-modulatory serotonergic system [60]. In line with the scope of this review, it is worth mentioning that 5-HT_4_Rs activation increases hippocampal outflow of Ach [61,62] (Table 1, Figure 2). First observed ex vivo in guinea pig hippocampal slices, this boosting effect of 5-HT_4_Rs agonists on Ach release was also identified in vivo and was interestingly found to be specific to the memory process [61].

#### 2.4.4. The Serotonergic System

Among monoaminergic systems, the serotonergic system is the most projected system in the brain. Mainly originating from dorsal and median raphe nuclei (DRN and MRN), serotonergic neurons send projections to the hippocampus [63]. All seven 5-HT receptors (5-HTRs) subfamilies are expressed in the hippocampus, each having a unique distribution pattern, although pattern overlapping is also observed [64,65].

The main supporting evidence for the involvement of 5-HT in memory function comes from observations of memory impairment after 5-HT depletion in human and animal model studies [66]. Numerous studies demonstrated the modulatory function exerted by the serotonergic system in memory function either in animal models or in human, both in physiological and in pathological aging condition [67].

As stated earlier, the identification of 5-HT_4_Rs on hippocampal glutamatergic neurons [51] strongly supports an interplay between the serotonergic and the glutamatergic system that could undoubtedly benefit memory function. Hence, the serotonergic system appears to be central to memory function in that it has intimate interactions with both the two major neurotransmission systems and other neuromodulator systems [47].

## 3. Relevance of 5-HT_4_Rs Modulation in Memory Disorders

5-HT_4_Rs belong to excitatory Gαs (stimulatory alpha subunit) protein-coupled receptors (GPCR). Their activation exerts a stimulatory effect through the activation of adenylate cyclase (ADC) as a primary mode of signal transduction on cAMP concentration. This second messenger interacts with various other proteins including PKA, which is known to modulate the activation of gene expression modifying transcription factors, such as the cAMP response element-binding protein (CREB) [68]. Additionally, an intriguing aspect of metabotropic 5-HTRs is their ability to elicit non-canonical pathways that can be G-protein independent. With regard to 5-HT_4_Rs, their activation can initiate phosphorylation of their associated non-receptor tyrosine kinase Src, which activates mitogen-activated protein kinases (MAPK) including the extracellular signal-regulated kinases (ERK) [68,69]. Quite interestingly, these molecular actors also appear to be involved in LTP. Moreover, cAMP/signaling and BDNF expression were found to be disrupted in a number of animal models of neurological disorders [70,71] and found to be enhanced after 5-HT_4_Rs activation [70,71] (Figure 2). Altogether, this raises the interest of 5-HT_4_Rs-targeting in plasticity-related memory enhancement.

### 3.1. Insights from Animal Behavior Investigations

The idea that 5-HT_4_Rs agonists are promising drug candidates for memory impairments—especially those related to hippocampal dysfunction—was firstly supported by behavioral studies on different animal models [72,73]. On one hand, cognitive impairments were often reported following antagonism (either pharmacologic agent or optogenetic construct) of 5-HT_4_Rs [74]. Surprisingly, the genetic ablation of 5-HT_4_Rs did not alter learning and memory capacities in mice. However, the deleterious effect of scopolamine (a cholinergic antagonist) on long term memory was enhanced in 5-HT_4_Rs KO mice [75]. On the other hand, a very large number of preclinical studies reported consensual data supporting the beneficial effects of 5-HT_4_Rs activation on memory performance. Overall, administration of 5-HT_4_Rs agonists increased the learning rate in a hippocampus-dependent spatial task, such as the MWM [76] and the object recognition test [77,78,79]. 5-HT_4_Rs agonists also restored memory impairments in animals treated with cholinergic antagonists [80,81,82], in aged animal [77,83] and in transgenic models of neurological diseases [84,85]. Additionally, it was recently reported that intra-hippocampal injection of a 5-HT_4_Rs agonist reduced sleep deprivation-induced memory impairments [86]. These behavioral effects of 5-HT_4_Rs modulation were extensively reviewed [73,87]. Likewise, chronic 5-HT_4_Rs activation was found to counterbalance learning and memory deficits induced by stress-induced depression [3].

Additionally, 5-HT_4_Rs have also been considered as an associative target of choice. Indeed, given the multidimensional and complex aspect of the pathogenesis of memory disorders, a new approach has emerged that consists of the simultaneous modulation of more than one target. After having proved the efficacy of 5-HT_4_Rs stimulating activity in co-administration protocols with different AchE inhibitors [78,88], the first multi-target drug ligand (MTDL) associating both activities has been designed. Named as Donecopride, this drug candidate was mainly developed for application in the field of AD [89]. Indeed, these promising results argue for the development of other MTDLs combining 5-HT_4_Rs agonistic activity with a different secondary target (other than AchE inhibitor) to be used for different medical application [90].

These observations constitute the first line of evidence for an interest in 5-HT_4_Rs activation in disorders related to hippocampal dysfunction. However, a limitation of preclinical research has certainly been the lack of investigation of 5-HT_4_Rs’ functional and/or expression alteration in animal models that display memory deficits [91]. In order to clarify if 5-HT_4_Rs changes are causative or involved in the etiology of diseases, their expression pattern needs to be assessed on a cellular level in preclinical models.

### 3.2. Distribution of 5-HT_4_Rs in CNS and Memory Disorders

The distribution of 5-HT_4_Rs within the brain is mainly restricted to the limbic system, thus intimately tied to memory function. The highest 5-HT_4_Rs mRNA levels and densities are found in caudate, putamen, accumbens, and in the hippocampal formation [92,93,94]. Within the hippocampal formation, the highest expression is found in the granule cell layer of the DG, followed by the pyramidal cell layer of the CA. Further, 5-HT_4_Rs exhibit a layered distribution within CA subfields, with the highest densities identified in the stratum oriens and stratum radiatum. This suggests a localization of receptors at both basal and apical dendritic fields of pyramidal cells. Radio-ligand assays also show strong labelling in the stratum lucidum of the CA3 area, probably reflecting the presence of 5-HT_4_Rs on MF [95,96].

Ligand binding studies also help to reinforce the idea that 5-HT_4_Rs play a pivotal role in memory function. In fact, the hippocampal density of 5-HT_4_Rs was found to be inversely correlated with episodic memory test performance in healthy subjects [97]. Further, it has also been observed that a striking feature of aging is the dramatic decrease in 5-HT_4_Rs density that occurs [93,98]. Likewise, the loss of 5-HT_4_Rs expression was also observed in different cohorts of patients suffering from memory deficits [91,99] and was correlated with the stage of the disease. For instance, a post-mortem brain analysis in AD patients reported a 70% decrease in hippocampal 5-HT_4_Rs [100], a change that was positively correlated to amyloid beta peptide load [98]. Additionally, reduced 5-HT_4_Rs binding was observed in the hippocampus in an animal model of depression [101] (Figure 2).

Moreover, it has been proposed that improvement of memory performance in patients who suffer from memory disorders is supported by up-regulation of 5-HT_4_Rs, which in turns stimulates hippocampal 5-HT release as shown in rodents [102,103] (Table 1, Figure 2). Indeed, there is now a large body of preclinical data showing a dynamic positive correlation between central 5-HT levels and 5-HT_4_Rs densities. For instance, 5-HT_4_Rs KO mice have diminished tissue levels of 5-HT (and its main metabolite, 5-HIAA) [104]. Hence, 5-HT_4_Rs activation could enhance 5-HT global tone through the positive feedback loop projecting from the prefrontal cortex to the DRN and thus, to the hippocampus [91]. If so, this could account for the variation of 5-HT_4_Rs expression observed in AD. Indeed, an upregulation of 5-HT_4_Rs expression occurs at the pre-clinical stage of the disease and continues along with dementia progressing (up to mild stage), as if a compensatory strategy was put in place (in response to decrease in interstitial 5-HT levels), until exhaustion [98]. Indeed, the loss of serotonergic cells in AD patients can reach above 70% in the DRN and MRN [105] and can even be reduced to undetectable levels [106,107]. This ultimately contributes to a decrease in hippocampal 5-HT neurotransmission, which has been identified as a correlate of cognitive impairment [108] (Figure 2). Altogether, the changes in 5-HT_4_Rs density may reflect the abnormal range of 5HT levels required for memory functioning. Hence, the clinical stage of the disease during which 5-HT_4_Rs may be used appears critical.

### 3.3. Morphological/Structural Alterations of Hippocampal Formation in Memory Disorders

Although a host of brain changes are likely to be responsible for cognitive decline, structural and functional hippocampal alterations were identified as one major correlate. Therefore, magnetic resonance imaging (MRI) scan has become one of the most common markers associated with cognitive scales performed in aging studies or in clinical practice to measure brain disease burden [109]. Whilst hippocampal atrophy is an important imaging correlate of memory impairments observed in numerous brain disorders, its pattern of alteration may vary according to the disease and the stage of the disorder.

For instance, within hippocampal formation, the EC appears to be most resistant to the effects of normal aging, as changes are mainly restricted to the DG and CA3. In contrast, the EC is most vulnerable to AD while the DG and CA3 remain relatively preserved. With regard to the CA1 area and the subiculum, they are mainly affected in SCZ and MDD respectively. Unlike AD, no prominent cell loss has been identified in aging, SCZ and MDD, suggesting rather, functional alterations such as connectivity dysfunction [109]. Consistently, an MRI-based study using diffusion tensor imaging to detect dendritic integrity revealed age-related alterations of DG and CA3 dendrites in aged patients [26] (Figure 2). Nevertheless, the measure of hippocampal volume was found to be sensitive enough to aging and to neurodegenerative and psychiatric disorders. For instance, after the age of 70, total hippocampal volume is believed to decrease at a rate of ~1.5% a year [110]. Additionally, hippocampal volume loss has been shown to reach 10 to 15% in mild cognitive impaired (MCI) patients [110]. Patients suffering from schizophrenia, PD or depression also exhibit hippocampal volume reduction of 4–6% relative to healthy subjects [111,112,113].

Of most interest, several lines of evidence now support that 5-HT_4_Rs agonists could limit such hippocampal deterioration at different levels, notably in AD context (see Table 1 for extensive details).

First, the above reported hippocampal volume loss—either due to aging or pathological condition—can be compensated, at least partly, through neurogenesis boost, which is altered in various neurological and psychiatric diseases [114]. However, it has been shown that sub-chronic treatment with 5-HT_4_Rs agonists induced an increase in BDNF expression in the CA1 (72%) as well as in the DG (52%), this latter demonstrating a neuro-proliferative activity [70]. Further, increased levels of other neurotrophic factors have also been reported after 5-HT_4_Rs agonist treatment, such as the soluble (non-amyloidogenic) form of the amyloid precursor protein alpha (sAPPα) (Table 1, Figure 2). The functions of sAPPα include—but are not limited to—proliferation, neuroprotection, synaptic plasticity, memory formation, neurogenesis and neuritogenesis in cell culture and animal models. Quite interestingly, sAPPα production was found to be promoted following acute [115] and chronic 5-HT_4_Rs activation in various conditions that include cell lines overexpressing 5-HT_4_Rs (50% increase) [61,116,117,118,119] as well as neuroblastoma cell line [61], and cultured neurons from a mouse model of AD [85,120,121,122]. A similar effect was observed in vivo both in healthy mice (2-fold increase) [115] and in AD mice models (1.5-fold increase) [84,85]. In the context of AD, the effects of 5-HT_4_Rs activation on sAPPα production would confer an additional benefit though a reduction in amyloid load (31–55% in a mouse model of AD [84,85]) by limiting the amyloidogenic pathway. Indeed, accumulation of neurotoxic Aβ in key hippocampal regions appears to be the primary cause of neuronal death leading to hippocampal atrophy [123] (Table 1, Figure 2).

Second, additional data supporting the putative role of 5-HT_4_Rs in preserving hippocampal integrity come from studies focusing on dendritic spines hosting excitatory synapses. The latter are dynamic structures, whose formation, shape, volume and collapse depend on neural activity. Therefore, they influence (but also can in return be influenced) the learning processes and memory performance [18]. In mice, pharmacological activation of 5-HT_4_Rs was shown to selectively potentiate the learning-induced dendritic spines’ growth (+6%) within the hippocampal CA1 (Table 1, Figure 2). This was not found in other brain structures that are not as much implicated in memory processing (i.e., primary visual cortex) [69]. Moreover, in a recent study using high resolution time lapse FRET imaging on neuronal dendrites, 5-HT_4_Rs activation was found to prompt maturation of synaptic connections via the 5-HT_4_R/G13/RhoA signaling cascade [124]. By activating PKA and BDNF/TrkB signaling pathways, 5-HT_4_Rs activation also promoted total dendritic length, number of primary dendrites and branching index in vitro [125]. Since spines represent potential sites of postsynaptic excitatory input, boosting their growth and maturation may translate into an increase in the number of excitatory synapses.

Finally, it is worth mentioning that reactive astrocytes are found both in human AD patients and AD mice models. Post-mortem morphological brain studies demonstrate close interaction between astrocytes and Aβ deposition in AD patients. In fact, reactive astrocytes are thought to be involved in Aβ production by upregulating β-secretase activity and APP in the diseased brain [126]. In this way, any strategy that would participate in a reduction in astrogliosis may substantially contribute to a reduction in Aβ load and subsequent neuronal loss. IL-1β and MCP-1 are two key pro-inflammatory mediators involved in glial reactivity whose levels have been found to be reduced by 30% to 45% following chronic 5-HT_4_Rs activation in an early onset mouse model of AD [84] (Table 1). Consequently, astrogliosis and microgliosis were reduced by 50–60% and 57% respectively in the EC, an area of the hippocampal formation that is particularly susceptible to degeneration in AD, as previously discussed [84,85]. Of note, astrogliosis reduction was even more pronounced with a longer duration of 5-HT_4_Rs agonist treatment [85]. Hence, 5-HT_4_Rs modulation could modify AD pathogenesis by targeting inflammatory pathways in glial cells.

The demonstration of such beneficial effects of 5-HT_4_Rs ligands holds promise for the development of disease-modifying drugs, which represents a yet unmet medical need. Of course, upstream correction of the pathological drivers of the disease is crucial to significantly improving the downstream symptoms and to prevent progressive cognitive deterioration. To date, preclinical studies that showed beneficial effects of 5-HT_4_Rs on hippocampal function have been mainly performed in either non-pathological conditions or in experimental models of the disease (cell lines or animal models). However, it seems important to stress that the pathology of AD shares a number of hippocampal alterations with ageing, SCZ, MDD and PD as discussed above. This ultimately raises the hope for potential translation of such beneficial effects of 5-HT_4_Rs in a large number of brain diseases.

### 3.4. Functional Synaptic Plasticity Impairments

Considered as the cellular support of memory, LTP has received much attention in the search for a better understanding of the mechanisms involved in memory disorders. Veritably, impairment of hippocampal synaptic function is often considered as an early detectable feature of aging and/or pathological stage, well before the first memory symptom appearance or before the observation of hippocampal atrophy.

Downregulation of plasticity-related proteins such as cAMP and CREB have, for instance, been observed in the hippocampus of both animal models of AD, and AD patients [127] (Table 1). In this regard, there is accumulating evidence for a beneficial action of 5-HT_4_Rs agonists on cAMP/CREB signaling. Consistently, increases in both cAMP and CREB levels as well as the phosphorylated form (active form) of CREB (pCREB) were found both in healthy rats [70] and the neuroblastoma cell line [121] following 5-HT_4_Rs activation.

Interestingly, such effects have been investigated for the first time in a mouse model of PD. In this study, the cAMP and pCREB levels were found to be increased in the DG following an acute treatment with 5-HT_4_Rs agonist and correlated to facilitation of memory performance [71].

Additionally, recent technical developments using repetitive Transcranial Magnetic Stimulation (rTMS) and ElectroEncephaloGraphy (EEG), has enabled the non-invasive investigation of LTP in human cortical tissue [43,128]. Thus, a decrease in LTP-like plasticity was observed in various conditions, including aging [129], MDD [130,131], SCZ [132,133] and AD [134] (Figure 2). Although based on a cortical readout, the conclusions drawn are overall consistent with those coming from the deep electrophysiological recordings, conducted in different animal models. Indeed, despite a few discrepancies (mainly related to differences in protocols used, such as animal species, strain, sex, electric conditioning stimulation), most preclinical studies reported an impaired hippocampal synaptic plasticity [135,136,137,138]. Those alterations could be specific to the condition investigated (aging, model of neurodegenerative disease such as AD), and also of the hippocampal subfields targeted (DG, SC-CA1 pathway).

However, the effects of 5-HT_4_Rs modulation on synaptic plasticity have been little studied, with only eight studies performed between 2001 and present, and results varying according to the hippocampal subfield investigated (Table 2).

The sole study which investigated the subiculum plasticity did not show any change of in vivo LTP following 5-HT_4_Rs activation [139]. Conversely, regarding DG and CA3 plasticity, all three in vivo studies reported an impaired time-course of LTP, which returned to baseline levels after 5-HT_4_Rs activation [140,141,142]. Finally, regarding hippocampal CA1 subfield, conflicting results were reported. Indeed, the first research group reported an enhanced in vivo LTP after intracerebroventricular (icv) injection of 5-HT_4_Rs agonist [82]. Additionally, this enhancement of LTP magnitude was blocked either by 5-HT_4_Rs antagonist or scopolamine. Conversely, the second research group did not observe any change of in vivo LTP. However, the icv administration of 5-HT_4_Rs agonist fully blocked learning-induced depotentiation of LTP [143], therefore suggesting a role for 5-HT_4_Rs in behavioral meta-plasticity. Interestingly, an electrophysiological experiment was recently conducted ex vivo on a hippocampal slice to investigate the effects of 5-HT_4_Rs activation on synaptic plasticity [57]. Opposite results were again observed ex vivo, but here they were linked to the frequency stimulation used to induce LTP. Thus, LTP was found to be specifically impaired after θ-burst, but not γ-burst. Within the hippocampus, the interaction between γ and θ rhythmic activities is critical for memory formation and the two experimental protocols impact network activity differently. Indeed, contrary to γ-burst, θ-burst efficacy of induction mainly relies on fine regulation of GABAergic neurotransmission, through notably a disinhibition process mediated by GABA auto-receptors [36,144]. While strengthening the theory of a tight interplay between 5-HT_4_Rs and the GABAergic system [55,56,57] these results argue in favor of targeting the 5-HT_4_Rs to treat memory disorders. Indeed, altered GABA neurotransmission—and the corollary imbalance between excitatory and inhibitory neurotransmission—has been repeatedly reported in many memory–neural circuit disorders [52] (Figure 2).

Finally, it should be noted that 5-HT_4_Rs agonists prevent persistent LTD in all subfields of hippocampal formation (CA3, DG and CA1) [80,143] with the exception of one study which reported an increase [139] (Table 2). In the same way, 5-HT_4_Rs agonists prevent DP in the DG [142] (Table 2).

Overall, the aforementioned set of data seems difficult to reconcile with the conventional view that increased LTP amplitude correlates with improved memory. However, literature data conversely suggest that stimulation of 5-HT_4_Rs may reset plasticity to a baseline level, rather than potentiate or reduce the synaptic strength. In the same way, it has been proposed that LTP decays may reflect a reset in hippocampal circuits back to a certain level, so that new information can be more effectively processed later [145]. This is based on the principle of homeostatic plasticity whereby network excitability is comprised of uncompensated LTP and LTD. Indeed, either insufficient or excessive synaptic plasticity prevents learning and memory formation [146]. The key feature of this popular model lies in synaptic gain adjustment: prolonged increase in activity downscales synapses to maintain an overall average firing rate, and vice versa [147].

In other words, the above discussed data support the fact that 5-HT_4_Rs, through their modulatory effects on synaptic plasticity processes, will enable the hippocampus to ensure its filtering role of information during acquisition and more variable changes in the downstream areas. This perspective seems consistent with clinical data that suggest that an increased signal-to-noise ratio within the hippocampus improves the encoding accuracy, a function which is thought to be mainly supported by the DG where 5-HT_4_Rs are most abundantly expressed [148]. Overall, this is based on the core idea that the hippocampus yields a limited storage space, where relevant information is temporarily stored. The storage process is then triggered whenever the environment configuration is significant [24,148]. Hence, at a network level, it is reasonable to think that it might be beneficial to reduce excitability in regions that are primarily involved in information filtering, such as DG-CA3, to make any new event more salient. Further, a higher activation level in the DG/CA3 hippocampal region was reported both in MCI patients and in animal models of age-related memory loss. Disruption of this hyperactivity by pharmacological manipulations was associated with an improvement in cognitive function [149]. Interestingly, most electrophysiological studies consent on increased excitability of hippocampal pyramidal cells following 5-HT_4_Rs activation. Indeed, activation of ADC also leads to potassium channel inhibition and subsequent reduction in neurons after hyperpolarization [73]. Hence, 5-HT_4_Rs agonists were found to enhance population spike amplitude in CA1 hippocampal slices, both in healthy animals [150,151] and in a mouse model of AD [151]. Recent work also suggests a regulatory role of 5-HT_4_Rs in GC cells’ excitability [152]. At the cellular level, 5-HT_4_Rs activation results in tonic depolarizing currents [124]. Taken altogether, 5-HT_4_Rs activation beneficial effects would appear to be two-fold. First, it would preserve the excitatory/inhibitory (E/I) balance, through either direct or indirect modulation of GABAergic neurotransmission. Second, 5-HT_4_Rs would transiently exert a boosting effect on synaptic efficiency, through a selective increase in neurons’ ability to fire action potentials whenever the incoming input is strong enough to alleviate inhibition.

Moreover, according to the gating hypothesis which suggests that levels of activity are transferred thorough the hippocampus, a high degree of CA3 activation will provide strong inhibitory inputs to CA1. In contrast, small fluctuations in CA3 activity will not provide sufficient excitation to bring enough CA1 neurons above activation threshold [24]. This raises the important need of evaluating the effects of 5-HT_4_Rs activation on synaptic plasticity within the different hippocampal subfields simultaneously.

### 3.5. 5-HT_4_Rs in Clinical Trials

The patent applications relating to 5-HT_4_Rs modulators were very recently the subject of a literature review [9]. Among the most promising 5-HT_4_Rs agonists was SL65.0155 (also called Caperserod) developed by Sanofi-Aventis. Despite its encouraging results in the preclinical field [83,153], to the best of the authors’ knowledge, this compound did not reach clinical trials (as it is not referenced in ClinicalTrials.gov data base).

To date, four 5-HT_4_Rs partial agonists have been tested in clinical trials to treat memory disorders. As such, VRX-03011 (also called PRX-03140) from Epix Pharmaceuticals (NCT00672945) [154] reached the milestone safety and proof of concept phase (phase 2b) of clinical trials for the treatment of AD. However, since then, no additional information appears on the clinical trials website and no original papers have been published, suggesting that research on this compound has been discontinued. In 2011, after demonstrating encouraging results in pre-clinical investigations, PF-04995274 (Pfizer, NCT03516604) reached phase 1 of clinical trials against cognitive impairment in AD, but has shown limited blood brain barrier permeation [9]. Quite recently, SUVN-D4010, a novel, potent, highly selective 5-HT_4_Rs partial agonist intended for the treatment of cognitive disorders, was found to be safe and well tolerated in healthy human subjects, even in elderly population (Suven Life Sciences, NCT02575482 and NCT03031574). Lastly, the results published last year regarding prucalopride are also of high interest. Indeed, while already approved by the FDA in 2018 to treat chronic idiopathic constipation, prucalopride was investigated in a battery of cognitive tests related to hippocampal functions. In healthy human subjects, prucalopride showed beneficial effects on learning and memory performance (NCT03572790) [148] and is currently under investigation for its role in depression. Evidence for improved memory performance after 5-HT_4_Rs activation in humans was extended by a very recent fMRI study. Following prucalopride intake, hippocampal activity during memory recall was significantly increased compared with volunteers receiving a placebo [155].

Overall, arguments to consider 5-HT_4_Rs as a target of choice for the treatment of memory impairments mainly stem from preclinical evidence. In fact, only a few experiments were performed on humans. Therefore, beyond the encouraging results of preclinical studies, it is wise to be cautious when editing conclusions because the touchy step of “translation from bench to bedside” often holds many disappointments. The human and rodents’ hippocampus display quite common structural anatomy and play a similar function in memory process. However, memory function (and so its integrity) relies on several distributed regions in the whole brain that conversely may display striking difference across species (notably cortical regions). For instance, in the model of hippocampal disengagement of long-term memory [156] the hippocampus would be crucial for recent memory retrieval, while cortical areas would play a key role for remote memory retrieval. 

## 4. Concluding Remarks and Future Perspectives

By spanning key aspects of hippocampal alterations that pave the way to decline in memory function, this review draws an original outline of the interest of 5-HT_4_Rs targeting for memory impairment.

The use of 5-HT_4_Rs ligands in the treatment of memory deficits is still an ongoing challenge but has long been—and still unfortunately is—restricted to AD and MDD. However, as highlighted in this review, a number of functional and morphological changes within the hippocampus are a common denominator of a broader range of both normal ageing and neurological diseases (such as PD, MDD, SCZ). A large amount of data from both animal models and humans have now reached a consensus on the fact that 5-HT_4_Rs activation can attenuate some of these hippocampal alterations. This ultimately raises the exciting potential of restoring—or at least limiting—memory decline in these pathologies. Nevertheless, a deeper understanding of the mechanisms at work is still needed and would help further development. In this view, studies that investigate 5-HT_4_Rs effects on hippocampal function in a more integrated view should provide substantial insights. This constitutes an interesting framework for the authors’ current research that recently revealed a modulatory effect on HFS-induced LTP, measured ex vivo after in vivo administration of a 5-HT_4_Rs agonist (unpublished data, [157]). These changes were accompanied with variations in the levels of the hippocampal neurotransmitter highly involved in memory function and associated synaptic plasticity.

## Figures and Tables

**Table 1:** Summary of pathological drivers of hippocampal atrophy contributing to memory impairment in AD pathology and beneficial effects of 5-HT_4_Rs ligands. Abbreviations: Dose/Con.: dose/concentration; mg/kg.d: mg/kg per day; ↑ denotes an increase; ↓ denotes a decrease; Aβ: beta-amyloid peptide; Ach: acetylcholine; AD: Alzheimer’s disease; APP: amyloid precursor protein; APP cleaving enzyme 1; Bace-1: beta-site; cAMP: cyclic adenosine monophosphate; CREB: cAMP response element-binding protein; EC: entorhinal cortex; GABA: gamma-aminobutyric acid; icv: intracerebroventricular; IL-1β: interleukin 1 beta; IPSPs: inhibitory postsynaptic potentials; MCP-1: monocyte chemoattractant protein-1; MMP-9: matrix metalloproteinase 9; NA: not applicable; pCREB: phosphorylated cAMP response element-binding protein; PD: Parkinson disease; sAPPα: soluble alpha-amyloid precursor protein; 5-HIAA: 5-hydroxyindoleacetic acid; 5-HT: serotonin; 5-HTR: serotonin receptor.

Alteration	Contributing Factor	5-HT_4_Rs Agonist	Dose/Con.	Treatment Duration	Preclinical Model	Target Brain Disease	Outcome of 5-HT_4_Rs Activation	References
	Aβ-mediated cell death (Dysfunction in APP metabolism)	*VRX-0311*	0.1 nM–10 µM	30 min	CHO cells stably expressing the human 5-HT_4(e)_ receptor and APP695	AD	Concentration-dependent ↑ sAPPα	Mohler et al. 2007
		*Prucalopride*	1 µM	2 h	HEK-293 expressing SEAP-tagged APP and 5-HT_4_Rs	AD	↑ sAPPα secretion (50%) through stimulation of α-secretase	Cochet et al. 2013
		*Prucalopride*	1 µM	30 min	CHO cell line expressing sAPPa and 5-HT_4_Rs	AD	↑ sAPPα secretion	Lezoualc’h and Robert, 2003 Robert et al. 2001
Hippocampal volume loss		*SSP-002392*	5 mg/kg	26—37 days	APP/PS1 mice (4–5 and 12 months)	AD	↓ soluble and insoluble hippocampal Aβ40 and Aβ42 ↓ total number of Aβ deposits in mice aged 4–5 months ↓ Bace-1, Adam17 (50%) and Nicastrin ↑ astrogliosis and microgliosis (Aβ degradation)	Tesseur et al. 2013
			10 nM		SH-SY5Y human neuroblastoma cell line	NA	↑ sAPPα release	
		*RS67333*	2 mg/kg		APP/PS1 mice (7–8 months)	AD	No change in Aβ	
		*RS67333*	3 µM	1 h, 2 h, 4 h, 8 h, 24 h and 48 h	H4/AβPP/5-HT_4_ cells	AD	↑ sAPPα production (102%, 265%, 343% of control at 8 h, 24 h, and 48 h respectively) through MMP-9 (role in α-secretase activity)	Hashimoto et al. 2012
		*RS67333*	3 mg/kg	10 days	Female Tg2576 transgenic mice (10–12 months)	AD	↓ in Aβ load (30%)	
		*RS67333*	-	30 min	COS-7 cells transiently expressing 5-HT_4_Rs and SEAP-APP	AD	↑ sAPPα release	Giannoni et al. 2013
		*RS67333*	1 mg/kg.d (twice a week)	3 months	5xFAD female mice	AD	↑ hippocampal sAPPα (1.5 fold) ↓ in Aβ load (hippocampus: 48%, EC: 55%) ↓ Aβ42 levels in the insoluble and soluble factions (33% and 53 % respectively)	
		*RS67333*	1 mg/kg.d (twice a week)	3 months	5xFAD female mice	AD	↓ in Aβ load in EC (31–33%)	Baranger et al. 2017
		*RS67333*	0.01µM-100µM	2 days exposure	Cortical Primary culture from Tg2576 mice	AD	Dose-dependent ↓ of Aβ levels 90–95% depletion of both Aβ40 and Aβ42 at 30μM Protection from Aβ-mediated cell death (increase in neuronal survival)	Cho and Hu, 2007
	Neuro-inflammation	*RS67333*	1 mg/kg.d	2 weeks	5xFAD male mice (4 months)	NA	↓ astroglial reactivity (61%) ↓ pro-inflammatory mediators IL-1β (25%) and MCP-1 30%) after 4 months’ treatment	Baranger et al. 2017
		*RS67333*	1 mg/kg.d (twice a week	3 months	5xFAD male mice	AD	↓ astrogliosis (49%) ↓ microgliosis (57%)	Giannoni et al. 2013
Network plasticity impairments	Synaptic loss and connectivity alterations	*SL65.0155*	0.01 mg/kg	4 days	Adult C57BL/6J mice	NA	Potentiates learning-induced spine growth (+6% relative to controls)	Restivo et al. 2008
		*BIMU-8*	10 µM	10 min	N1E-155 Neuroblastoma cells	NA	Boosts phosphorylation of cofilin (regulator of neuronal morphology and spinogenesis)	Schill et al. 2020
					Hippocampal primary culture from C57BL/6J mice	NA	Prompts dendritic spine maturation (increasing the number of active axo-spinous excitatory synapses in dendritic branches of principal neurons) Boosts numbers of excitatory synapses	
	↓ plasticity-related proteins	*Prucalopride* *Velusetrag*	1.5- 3 mg/kg 3 mg/kg	Single dose	(MPTP)-induced PD model mice	PD	↑ cAMP levels (with stronger effect of Velusetrag) ↑ pCREB positive cells in DG	Ishii et al. 2019
		*RS67333*	1.5 mg/kd.d	3–7 days	Adult male Sprague-Dawley rats	NA	↑ pCREB/CREB ratio	Pascual-Brazo et al. 2011
		*SSP-002392* *Prucalopride*	0.0001 -1 mmol/L 0.01–1 mmol/L		SH-SY5Y human neuroblastoma cells	NA	↑ cAMP production (with stronger effect of SSP-002392)	Tesseur et al. 2013
			
	Inhibitory vs. excitatory imbalance	*BIMU-8*	0.2–2 µM	45 min	Guinea pig hippocampal slices	NA	Ach-dependent increase in electrically-evoked GABA release at low concentration (0.2–0.4 µM) Ach-dependent inhibition of electrically-evoked GABA release at higher concentration (0.7–2 µM)	Bianchi et al. 2002
		*Zacopride*	10 µM	5 min	Guinea pig hippocampal slices	NA	↑ IPSPs	Bijak and Misgeld, 1997
		*VRX-03011*	1–5 mg/kg	Single dose	Adult male Long Evans rats	NA	↑ Ach outflow under mnemonic demand	Mohler et al. 2007
		*Renzapride*	1 mg/kg (systemic) 100 µM (icv)	Single dose	Adult female Wistar rats	NA	Concentration-dependent ↑ hippocampal 5-HT levels (200%)	Ge and Barnes 1996
		*RS67333*	1.5 mg/kg.d	3 days	Adult male Sprague-Dawley rats	NA	↑ 5-HT levels (73%) ↓ 5-HIAA levels (27%) (no effect of 5-HT_4_Rs agonist in acute conditions)	Licht et al. 2010
		*BIMU-8*	0.2–4 µM	5 min	Guinea pig hippocampal slices	NA	↑ Ach outflow after electrical stimulation (but not at rest)	Siniscalchi et al. 1999

**Table 2:** Compilation of electrophysiological investigations of synaptic plasticity in rodents after pharmacological 5-HT_4_Rs activation.↑ denotes an increase; ↓ denotes a decrease; = denotes no change. Abbreviations: CA1, CA3: cornus ammonis 1,3; DG: dentate gyrus; DP: depotentiation; HFS: high frequency stimulation; LTD: long term depression; LTP: long term potentiation; LFS: low frequency stimulation; SUB: subiculum; TBS: theta burst stimulation.

Method	Hippocampal Area	Plasticity	Conditioning Stimulus	5-HT_4_Rs Agonist	Effects of 5-HT_4_Rs Activation on Plasticity	Reference
In vivo	DG	LTP	HFS (200 Hz)	RS67333	↓	Kulla and Manahan-Vaughan
		LTP	HFS (200 Hz)	5-Methoxytryptamine	=	
		LTP	HFS (10 × 400 Hz)	RS67333	Transient ↑ and curtailed	Marchetti et al. 2004
		LTP	HFS (200 Hz)	RS67333	Curtailed	Twarkowski et al. 2016
		DP	LFS (5 Hz)	RS67333	Blocked	
		LTD	LFS (1 Hz)	RS67633	↓	
	CA3	LTP	HFS (4 × 100 Hz)	RS67333	↓	Twarkowski et al. 2016
		LTD	LFS (1 Hz)	RS67333	↓	
	CA1	LTP	HFS (5 × 400 Hz)	SC53116	↑	Matsumoto et al. 2001
		LTP	HFS (4 × 100 Hz)	RS67333	=	Kemp and Manahan-Vaughan 2005
		LTD	LFS (1 Hz)	RS67333	↓	
Ex vivo	CA1	LTP	HFS (1 × 100 Hz)	RS67333	=	Lecouflet et al. 2020
		LTP	TBS (4 × 5 Hz)	RS67333	↓	
	SUB	LTP	HFS (4 × 100 Hz)	RS67333	=	
		LTD	LFS (1 Hz)	RS67333	↑	Wawra et al. 2014

## Data Availability

Not applicable.
